# Detectability of hemodynamic oscillations in cerebral cortex through functional near-infrared spectroscopy: a simulation study

**DOI:** 10.1117/1.NPh.11.3.035001

**Published:** 2024-07-03

**Authors:** Letizia Contini, Caterina Amendola, Davide Contini, Alessandro Torricelli, Lorenzo Spinelli, Rebecca Re

**Affiliations:** a Politecnico di Milano, Dipartimento di Fisica, Milan, Italy; b Istituto di Fotonica e Nanotecnologie, Consiglio Nazionale delle Ricerche, Milan, Italy

**Keywords:** time-domain functional near-infrared spectroscopy, continuous-wave functional near-infrared spectroscopy, diffuse optics, resting-state, hemodynamic oscillations, brain

## Abstract

**Significance:**

We explore the feasibility of using time-domain (TD) and continuous-wave (CW) functional near-infrared spectroscopy (fNIRS) to monitor brain hemodynamic oscillations during resting-state activity in humans, a phenomenon that is of increasing interest in the scientific and medical community and appears to be crucial to advancing the understanding of both healthy and pathological brain functioning.

**Aim:**

Our general object is to maximize fNIRS sensitivity to brain resting-state oscillations. More specifically, we aim to define comprehensive guidelines for optimizing main operational parameters in fNIRS measurements [average photon count rate, measurement length, sampling frequency, and source-detector distance (SSD)]. In addition, we compare TD and CW fNIRS performance for the detection and localization of oscillations.

**Approach:**

A series of synthetic TD and CW fNIRS signals were generated by exploiting the solution of the diffusion equation for two different geometries of the probed medium: a homogeneous medium and a bilayer medium. Known and periodical perturbations of the concentrations of oxy- and deoxy-hemoglobin were imposed in the medium, determining changes in its optical properties. The homogeneous slab model was used to determine the effect of multiple measurement parameters on fNIRS sensitivity to oscillatory phenomena, and the bilayer model was used to evaluate and compare the abilities of TD and CW fNIRS in detecting and isolating oscillations occurring at different depths. For TD fNIRS, two approaches to enhance depth-selectivity were evaluated: first, a time-windowing of the photon distribution of time-of-flight was performed, and then, the time-dependent mean partial pathlength (TMPP) method was used to retrieve the hemoglobin concentrations in the medium.

**Results:**

In the homogeneous medium case, the sensitivity of TD and CW fNIRS to periodical perturbations of the optical properties increases proportionally with the average photon count rate, the measurement length, and the sampling frequency and approximatively with the square of the SSD. In the bilayer medium case, the time-windowing method can detect and correctly localize the presence of oscillatory components in the TD fNIRS signal, even in the presence of very low photon count rates. The TMPP method demonstrates how to correctly retrieve the periodical variation of hemoglobin at different depths from the TD fNIRS signal acquired at a single SSD. For CW fNIRS, measurements taken at typical SSDs used for short-separation channel regression show notable sensitivity to oscillations occurring in the deep layer, challenging the assumptions underlying this correction method when the focus is on analyzing oscillatory phenomena.

**Conclusions:**

We demonstrated that the TD fNIRS technique allows for the detection and depth-localization of periodical fluctuations of the hemoglobin concentrations within the probed medium using an acquisition at a single SSD, offering an alternative to multi-distance CW fNIRS setups. Moreover, we offered some valuable guidelines that can assist researchers in defining optimal experimental protocols for fNIRS studies.

## 1 Introduction

Understanding the intricate hemodynamic phenomena occurring in the human brain is crucial for advancing our knowledge of brain functions and related pathologies. The vasomotion, Mayer waves, neurovascular coupling, and influence of cardiac and respiratory activities on brain tissue are among the phenomena that underpin brain functionality.^1^ Previous studies have explored this field using functional magnetic resonance imaging (fMRI) and functional near-infrared spectroscopy (fNIRS) in the continuous-wave (CW) and frequency-domain (FD) modalities. These studies were conducted both on healthy subjects^2^^,^^3^ and in the presence of various pathologies, such as stroke,^4^ mild to severe cognitive impairment,^5^ acute and traumatic brain injuries,^6^^,^^7^ autoregulation dysfunctions,^8^^,^^9^ and autism.^10^ Multiple fNIRS connectivity studies were also performed.^11^^,^^12^

To the best of our knowledge, only two previous studies attempted to investigate resting-state brain oscillations making use of time-domain (TD) fNIRS. In the first study, by Themelis et al.,^13^ *in-vivo* measurements on human and piglet heads were conducted using FD and TD fNIRS systems to detect hemodynamic systemic oscillations within the cortical tissue. The authors presented preliminary results indicating that the modulation of the cortical signal at 830 nm due to the cardiac activity can be recorded with both techniques. However, no power spectral density (PSD) analysis was provided. In the second study, Kacprzak et al.^14^ employed the “moment” approach^15^ to extract information on the hemodynamic behavior of different regions within the probed tissue. The authors analyzed the 0th (intensity) and 2nd (variance) centralized moments of the recorded photon distributions of time-of-flight (DTOFs), the former mainly reflecting superficial extra-cerebral variations, and the latter mainly reflecting cerebral compartment variations. Then, they derived the PSD of the intensity and variance time series. This study involved two groups of subjects, both measured during rest: a control group consisting of healthy individuals and a second group comprising patients with severe neurovascular disorders. Notably, statistically significant differences between the two groups were observed when comparing the obtained power spectra. In addition, in both studies, no Fourier-domain analysis was performed on the hemodynamic parameters.

Although these investigations have shed light on certain aspects of cerebral hemodynamics, the unique strengths of TD fNIRS have yet to be fully explored. Because of the poor signal-to-noise ratio (SNR), most of the TD fNIRS instruments operate at an acquisition rate of <2  Hz. This is typically enough for monitoring task-related cortical hemodynamic responses, but not for other applications such as studying the resting-state oscillations that occupy wider frequency ranges (up to 5 Hz).^16^^,^^17^ Recently developed TD fNIRS devices address this primary limitation.^14^^,^^18^^,^^19^ The enhanced SNR achieved by these devices empowers researchers to delve deeper into the investigation of cerebral hemodynamic oscillations, unlocking new possibilities for understanding brain functionality. In addition, the performance of CW fNIRS in terms of depth-selectivity for this application remains largely unexplored. Simulations found in the literature were mainly designed to characterize the sensitivity of fNIRS to cerebral and extra-cerebral hemodynamics at different values of the source-detector distance (SDD), by evaluating the signal response to instantaneous perturbations imposed in the medium.^20^^,^^21^

In our study, we address these limitations by conducting numerical simulations to evaluate the effect of key operational parameters on the performance of both TD and CW fNIRS in detecting periodical oscillations in the probed tissue hemodynamics, aiming at assisting researchers in defining optimal experimental protocols tailored to their specific study objectives.

## 2 Methods

### 2.1 Forward Simulations

To generate the synthetic TD fNIRS signals, namely, a dataset of DTOFs, numerical simulations based on a forward diffusion equation model at different SDDs were implemented using home-made software.^22^ Two distinct geometries, a homogeneous medium and a bilayer medium, were used to mimic the geometric characteristics of the tissue under investigation while assessing different objectives. For both the homogeneous and the bilayer geometry, the instrument response function^15^ (IRF) was modeled as an ideal Dirac delta function, and to mimic real experimental conditions, noise following the Poisson distribution was added to each DTOF.

#### 2.1.1 Homogeneous medium

First, with the homogeneous medium, we studied the impact on the sensitivity of the technique to periodic perturbations of the optical properties of the medium of the main operational parameters: the SDD, the average total number of photons used to generate the DTOF (${\overset{¯}{N}}_{\mathrm{tot}}$), the measurement length ($T_{\mathrm{meas}}$), and the sampling rate ($f_{s}$). To limit the complexity of our model, we only considered oxygenated hemoglobin ($O_{2}\mathrm{Hb}$) and deoxygenated hemoglobin (HHb) as the main absorbing chromophores in human tissues in the wavelength range of interest, i.e., 600 to 900 nm. As for their baseline concentrations, we assumed the values of 30 and 20  μM, respectively.^23^ Sinusoidal perturbations with a fixed amplitude of 1% of the baseline value and fixed frequency of 1 Hz were imposed to create temporal variations in both hemodynamic parameters. The 1 Hz frequency was selected to mimic the effect of the cardiac muscle contraction on the probed tissue, a phenomenon previously observed in several CW fNIRS studies,^24^^,^^25^ and a relative amplitude of 1% was used to represent the minimum amplitude expected for physiological noise components from the previous literature.^24^^,^^25^ To account for the lack of consensus in the literature regarding the relative phase of oscillations of the same origin between chromophores or across different medium domains, a random phase shift was applied to each perturbation.^26^^,^^27^ Then, by exploiting the known absorption spectra of $O_{2}\mathrm{Hb}$ and HHb, the hemodynamic parameters were translated, by Beer’s law, in the absorption coefficient ($\mu_{a}$) at two wavelengths, 690 and 830 nm. The reduced scattering coefficient was set according to the empirical approximation of the Mie theory (${\mu_{s}}^{\prime }=a(\lambda_{0}/\lambda {)}^{-b}$), with $a=10.0\;{\mathrm{cm}}^{-1}$, b=1.0, and $\lambda_{0}=690\;\mathrm{nm}$. Finally, the solution of the diffusion equation for a 5 cm-thick, laterally infinite homogeneous slab with a 1.4 refractive index was used to calculate the dataset of DTOFs^28^ in the range 0 to 5 ns, with a time resolution of 8 ps. First, three values of ${\overset{¯}{N}}_{\mathrm{tot}}$ were considered: $10^{4}$, $10^{5}$, and $10^{6}$ photons (ph), while maintaining the other parameters fixed: $T_{\mathrm{meas}}=15\;\min$, $f_{s}=20\;\mathrm{Hz}$ (case H_$N_{\mathrm{tot}}$). Then, three values of $T_{\mathrm{meas}}$ were considered: 5, 10, and 15 min, while maintaining ${\overset{¯}{N}}_{\mathrm{tot}}=10^{6}\;\mathrm{ph}$, and $f_{s}=20\;\mathrm{Hz}$ (case H_$T_{\mathrm{meas}}$). Finally, three values of $f_{s}$ were considered: 5, 10, and 20 Hz, while maintaining ${\overset{¯}{N}}_{\mathrm{tot}}=10^{6}\;\mathrm{ph}$, and $T_{\mathrm{meas}}=15\;\min$ (case H_$f_{s}$). In these simulations, two SDDs, 1 and 4 cm, were used. An additional simulation was performed using SSD from 1 to 6 cm, with steps of 1 cm, with the other parameters fixed (${\overset{¯}{N}}_{\mathrm{tot}}=10^{6}\;\mathrm{ph}$, $T_{\mathrm{meas}}=15\;\min$, $f_{s}=20\;\mathrm{Hz}$) to better explore the effect of SSD (case H_SDD). In Table 1, all of the parameters used in each simulation case for the homogeneous medium (H_$N_{\mathrm{tot}}$-H_SDD) are summarized.

**Table 1:** Parameters used in each simulation case for the homogeneous medium.

Case	${\overset{¯}{N}}_{\mathrm{tot}}$ (ph)	$T_{\mathrm{meas}}$ (min)	$f_{s}$ (Hz)	SDD (cm)	Perturbation	
					Amplitude (%)	Frequency (Hz)
H_$N_{\mathrm{tot}}$	$10^{4}$, $10^{5}$, $10^{6}$	15	20	1, 4	1	1
H_$T_{\mathrm{meas}}$	$10^{6}$	5, 10, 15	20	1, 4	1	1
H_$f_{s}$	$10^{6}$	15	5, 10, 20	1, 4	1	1
H_SDD	$10^{6}$	15	20	1, 2, 3, 4, 5, 6	1	1

#### 2.1.2 Bilayer medium

The bilayer medium allowed for the evaluation and comparison of approaches for depth-selectivity enhancement, both in CW and TD fNIRS. This comparison aimed to assess the ability of the techniques to localize perturbations originating at different depths and effectively reject superficial extra-cerebral contributions. The bilayer medium is composed of two stacked cylinders with a radius of R=10  cm with a total thickness of 5 cm and a refractive index of 1.4. The superficial layer (UP) had a thickness of 1 cm, and the deeper layer (DW) had a thickness of 4 cm. Both layers had identical baseline hemoglobin concentrations and optical properties so the two layers are only identified by different and independent perturbations of the hemoglobin concentrations in time. The time duration of measurements $T_{\mathrm{meas}}=15\;\min$ and the sampling frequency $f_{s}=20\;\mathrm{Hz}$ were fixed based on the results obtained for the homogeneous medium (Sec. 2.1.1). The average total number of photon counts ${\overset{¯}{N}}_{\mathrm{tot}}$ was varied as before by considering three values of $10^{4}$, $10^{5}$, and $10^{6}\;\mathrm{ph}$ and two SDDs of 1 and 4 cm. Preliminary simulations investigated the effect of a single oscillatory component imposed in either the UP layer (case B_UP) or the DW layer (case B_DW) while keeping the concentrations in the other layer constant at baseline values. The same perturbation as in the homogeneous medium was used: an oscillation of both $O_{2}\mathrm{Hb}$ and HHb concentrations with a frequency of 1 Hz and an amplitude of 1% of the baseline value. In addition, we focused on evaluating the effects generated through the superimposition of multiple Fourier contributions simultaneously originating in the UP and DW layers. Four cases were considered:

• B_UPDW: oscillations at 1 Hz with an amplitude of 1% of the average value imposed in both the UP and DW layers
• B_Amp: oscillations at 1 Hz with an amplitude of 1% and 0.5% of the average value imposed in the UP and DW layers, respectively
• B_Freq: oscillations at 0.2 and 1 Hz, with an amplitude of 1% of the average value, imposed in the UP and DW layers, respectively
• B_AmpFreq: an oscillation at 0.2 Hz with an amplitude of 1% of the average value imposed in the UP layer, and an oscillation at 1 Hz with an amplitude of 0.5% imposed in the DW layer.

These simulations were repeated with the applied perturbations in-phase or out-of-phase. The solution of the diffusion equation for the bilayer geometry was used to calculate the dataset of DTOFs^28^ in the range 0 to 5 ns, with a time resolution of 20 ps to limit the computational time. Table 2 summarizes the parameters used in each simulation case when the bilayer medium was used (B_UP-B_AmpFreq).

**Table 2:** Parameters used in each simulation case for the bilayer medium.

Case	${\overset{¯}{N}}_{\mathrm{tot}}$ (ph)	$T_{\mathrm{meas}}$ (min)	$f_{s}$ (Hz)	SDD (cm)	Perturbation UP		Perturbation DW	
					Amplitude (%)	Frequency (Hz)	Amplitude (%)	Frequency (Hz)
B_UP	$10^{4}$, $10^{5}$, $10^{6}$	15	20	1, 4	1	1	—	—
B_DW	$10^{4}$, $10^{5}$, $10^{6}$	15	20	1, 4	—	—	1	1
B_UPDW	$10^{4}$, $10^{5}$, $10^{6}$	15	20	1, 4	1	1	1	1
B_Amp	$10^{4}$, $10^{5}$, $10^{6}$	15	20	1, 4	1	1	0.5	1
B_Freq	$10^{4}$, $10^{5}$, $10^{6}$	15	20	1, 4	1	0.2	1	1
B_AmpFreq	$10^{4}$, $10^{5}$, $10^{6}$	15	20	1, 4	1	0.2	0.5	1

### 2.2 Data Analysis

#### 2.2.1 Estimate of optical and hemodynamic parameters

For the simulations carried out using the homogeneous medium, the DTOFs were numerically time integrated to retrieve the measured light intensity, represented as the number of total photons detected within the measurement window, which can be considered an equivalent of the CW fNIRS signal.

When the bilayer geometry was considered, a time-windowing approach was used to exploit the depth-selective information encoded in the DTOFs. For our analysis, we used 10 time windows (gates), each with a width of 500 ps, covering the time window between 0 and 5 ns (full width of the largest curve) following the IRF temporal position. Successively, the diffusion equation solution for a bilayer medium^28^ was used as a model for photon migration in a home-made software^22^ for non-linear fitting (based on the Levenberg–Marquardt minimization algorithm^29^). After calculating the baseline optical coefficients $\mu_{a}$ and ${\mu_{s}}^{\prime }$ using a homogeneous model fit, the time-dependent mean partial pathlength (TMPP) method^30^ was used to estimate the variations $\Delta \mu_{a,j}$ from the baseline value from each DTOF, to extract the absorption coefficients ($\mu_{a}+\Delta \mu_{a,j}$) in the two layers, j=1 or 2, of the medium at every time point. The TMPP method, presented by Zucchelli et al.,^30^ is based on a time gating of the DTOF, which is used to estimate the time-dependent mean pathlength $L_{j}(t)$ traveled by photons in each domain j of the medium with high accuracy. Once $L_{j}(t)$ is known, the variations $\Delta \mu_{a,j}$ can be retrieved by inverting the following formulation of the time-resolved reflectance curve: $R(t)=R_{0}(t)e^{-\underset{j}{\sum }\Delta \mu_{a,j}L_{j}(t)}$. Finally, Beer’s law was used to derive the hemodynamic parameters from the absorption coefficient.^28^

To enable a direct comparison between TD and CW fNIRS data, the DTOFs were again integrated over the measurement window. The variations of hemoglobin concentrations for the CW fNIRS configuration were then calculated by means of the modified Lambert–Beer law.^28^^,^^31^ Increases or decreases in hemoglobin concentration were expressed relative to a baseline, calculated as the average intensity over the entire measurement. The depth-selectivity in CW fNIRS is generally implemented using multiple acquisition points at different SDDs and exploiting the relationship between the SDD and the photon penetration depth.^32^ Signals acquired with an SDD of 1 and 4 cm were thus compared in this phase.

#### 2.2.2 Frequency-domain analysis

The PSD of the intensity and hemodynamic signals were calculated using a uniform algorithm across all data, implemented in Matlab (Release 2022a, The MathWorks Inc., Natick, Massachusetts, United States). First, the signals were detrended using a third-order polynomial fit to remove the slow component.^10^^,^^26^ Then, the periodogram estimation of the PSD was performed by employing the Cooley–Tukey fast Fourier transform algorithm.^33^^,^^34^ Because our aim was to evaluate the PSD with the maximum frequency resolution without any variance reduction and all the data series met the stationarity requirements, no windowing was applied.

In Fourier-domain analysis, evaluating the significance of spectral peaks is crucial: to discern peaks attributable to underlying oscillatory phenomena from Poisson noise background, an ad-hoc contrast function was introduced. The contrast function C(f) was defined to compare the PSD amplitude at frequency f to the average amplitude of the PSD in a noise-only interval, ε

$$C(f)=\frac{\mathrm{PSD}(f)}{\varepsilon }.$$ (1)

In Sec. 3, to quantify the contrast value efficiently, it is expressed in decibels, as needed

$$C(f{)}_{\mathrm{dB}}=10\cdot \log_{10}(\frac{\mathrm{PSD}(f)}{\varepsilon }).$$ (2)

Due to the characteristics of our synthetic signals, the PSD average noise ε was typically calculated by averaging the PSD values for frequencies higher than 5 Hz. However, for the case $f_{s}=5\;\mathrm{Hz}$, a modification in the definition of ε was necessary, as the sampling rate did not permit reconstruction of the PSD for frequencies higher than 2.5 Hz. In this case, ε was calculated as the average value of the PSD in the range 2<f≤2.5  Hz to avoid interference from the second harmonic perturbation at 2 Hz, if present.

## 3 Results

### 3.1 Homogeneous Medium

In this section, the effect of the main operational parameters on the detected signal is investigated. The results are shown in terms of PSD of the intensity signal, equivalent to the CW fNIRS signal. Therefore, the obtained results are still valid for both TD and CW fNIRS.

#### 3.1.1 Effect of the average total number of photon counts ${\overset{¯}{N}}_{\mathrm{tot}}$

**Fig. 1:**
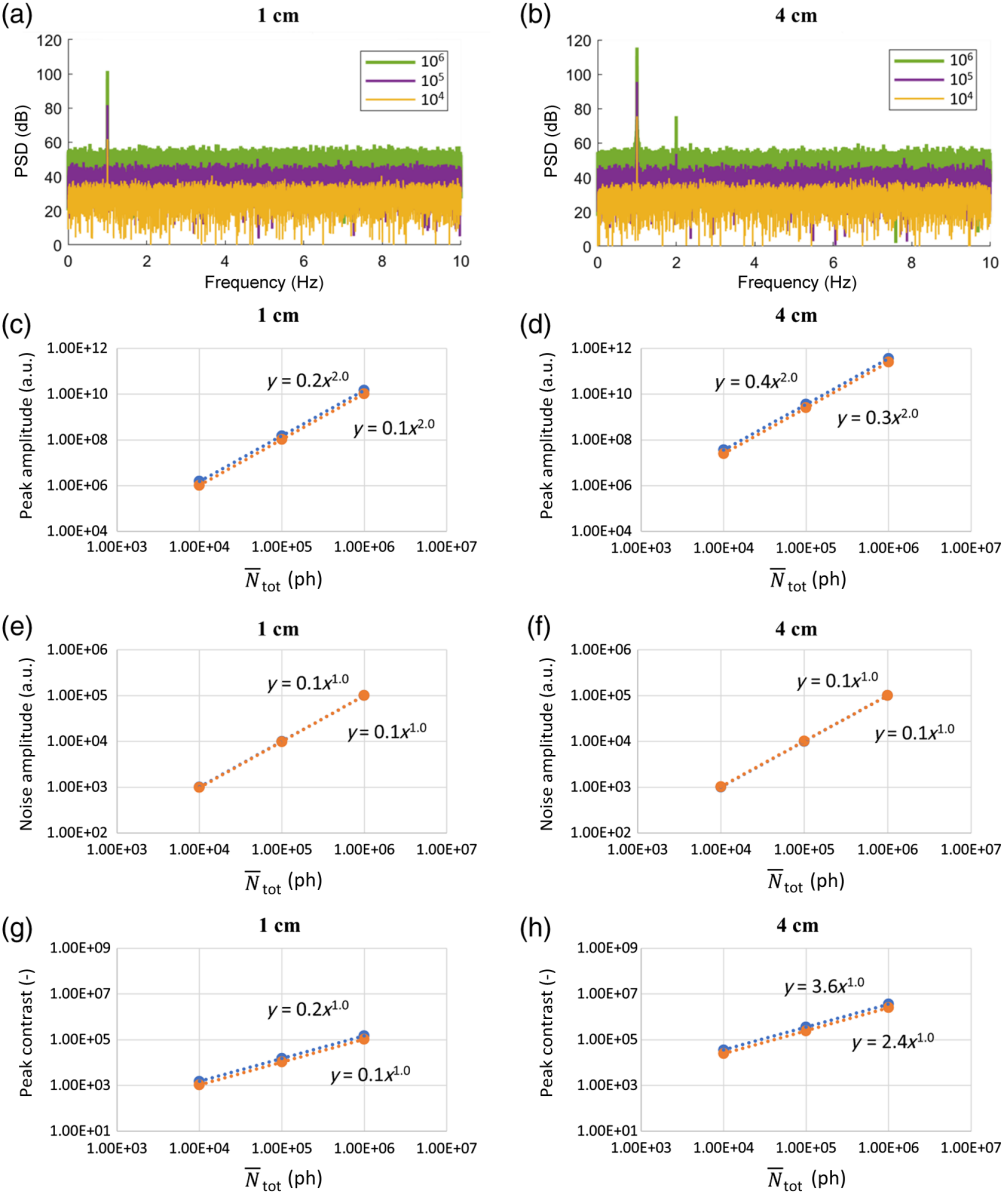
Effect of ${\overset{¯}{N}}_{\mathrm{tot}}$ (case H_$N_{\mathrm{tot}}$). The two columns represent signals acquired with SSD of 1 cm (left) and 4 cm (right). (a), (b) Examples of PSDs obtained at 690 nm. (c), (d) Amplitude of the spectral peak at 1 Hz; (e), (f) amplitude of the average noise; (g), (h) peak contrast. The results are reported for 690 nm, in blue, and 830 nm, in orange. A power law fit is applied to each dataset, all giving $R^{2}\geq 0.999$.

Figure 1 shows the effect of varying ${\overset{¯}{N}}_{\mathrm{tot}}$ on the spectra obtained in case H_$N_{\mathrm{tot}}$. Panels (a) and (b) show the obtained PSD for the SSD of 1 and 4 cm, respectively. Data are shown for a single wavelength (690 nm) as the results of the other wavelengths (830 nm) are similar. The peak at 1 Hz is clearly visible for every value of ${\overset{¯}{N}}_{\mathrm{tot}}$ (different colors). Increasing ${\overset{¯}{N}}_{\mathrm{tot}}$, higher amplitudes are obtained for the whole spectrum. Figures 1(c)–1(h) show the amplitude of the spectral peak at 1 Hz, the amplitude of the average noise, and the 1 Hz peak contrast as a function of ${\overset{¯}{N}}_{\mathrm{tot}}$ in logarithmic–logarithmic plots, for SSD = 1 cm (4 cm) and both wavelengths. A power law fit is applied to each data series, always returning an $R^{2}$ value higher than 0.999. It is interesting to note that a second harmonic of the 1 Hz peak (at 2 Hz) is visible for the longer SDD and the higher values of ${\overset{¯}{N}}_{\mathrm{tot}}$ [Fig. 1(b), in green and purple] but remains absent in the short SSD signals [Fig. 1(a)]. Comparing the difference in amplitude between the first and second harmonic (where visible) and assuming this difference to be consistent for all measurements, we confirmed that the second harmonic is covered by noise for all measurements in which it is not visible in the PSD. The results obtained by increasing ${\overset{¯}{N}}_{\mathrm{tot}}$ can be summarized as follows: (i) the 1 Hz peak amplitude increases with ${\left({\overset{¯}{N}}_{\mathrm{tot}}\right)}^{2}$; (ii) the average noise amplitude increases with ${\overset{¯}{N}}_{\mathrm{tot}}$; and (iii) the 1 Hz peak contrast value increases with ${\overset{¯}{N}}_{\mathrm{tot}}$.

#### 3.1.2 Effect of the measurement time $T_{\mathrm{meas}}$

**Fig. 2:**
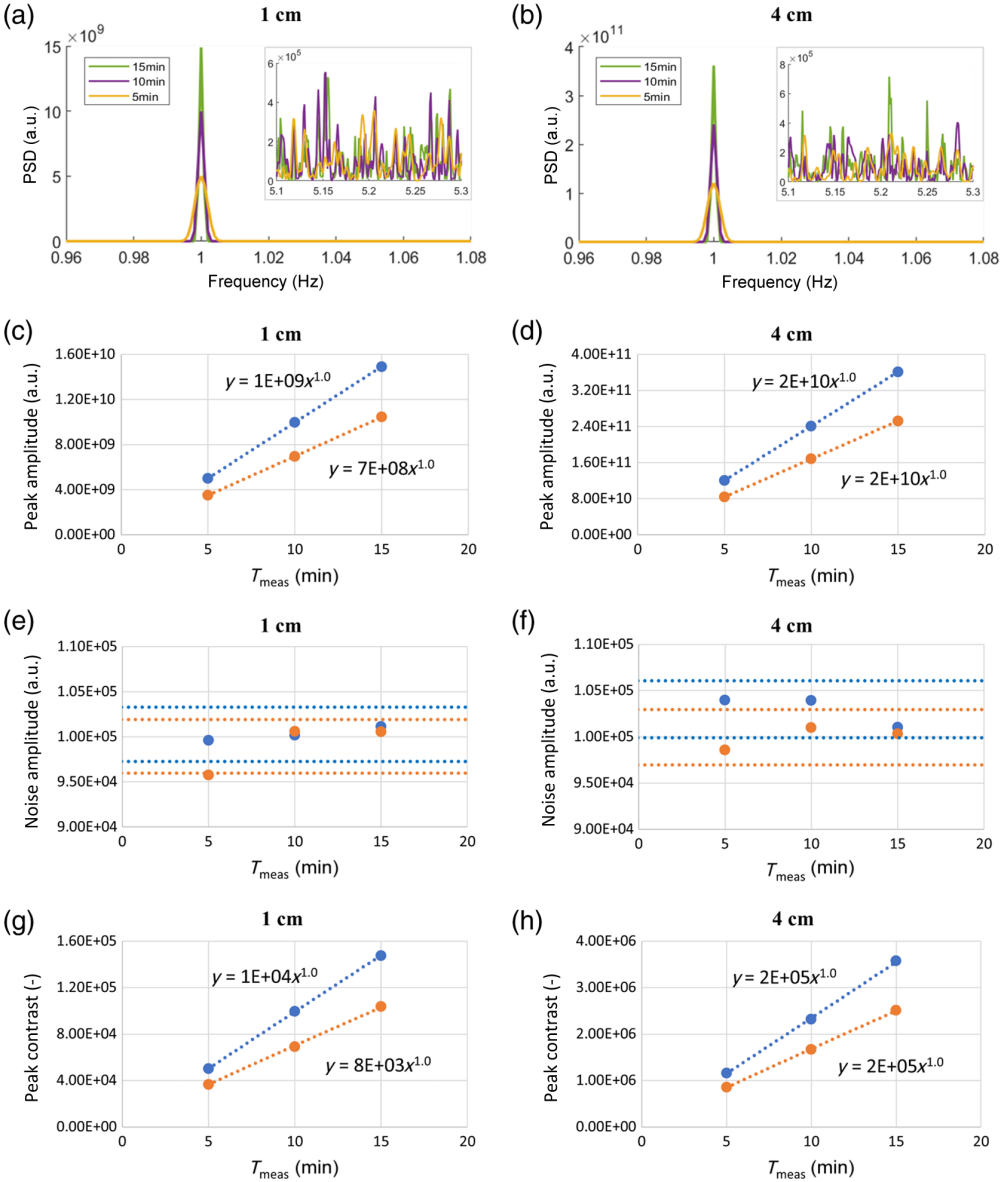
Effect of $T_{\mathrm{meas}}$ (case H_$T_{\mathrm{meas}}$). The two columns represent signals acquired with SSD of 1 cm (left) and 4 cm (right). (a), (b) Examples of PSDs obtained at 690 nm. The smaller boxes show a zoom of the PSD noise region. (c), (d) Amplitude of the spectral peak at 1 Hz; (e), (f) amplitude of the average noise; (g), (h) peak contrast. The results are reported for 690 nm, in blue, and 830 nm, in orange. A power law fit is applied to each dataset in panels (c), (d), (g), and (h), all giving $R^{2}\geq 0.999$. In panels (e) and (f), the horizontal lines represent a 3% variation around the mean value.

Figure 2 shows the effect of varying the $T_{\mathrm{meas}}$ on the spectra obtained in case H_$T_{\mathrm{meas}}$. Panels (a) and (b) show the PSD obtained for the SSD of 1 and 4 cm, respectively. Data are shown for a single wavelength (690 nm) as the results for the other wavelength (830 nm) are similar. The smaller box on the right of each graph represents a zoom on a higher-frequency region, showing the noise level in the three signals. Figures 2(c)–2(h) show the amplitude of the spectral peak at 1 Hz, the amplitude of the average noise, and the 1 Hz peak contrast as a function of $T_{\mathrm{meas}}$, for SSD = 1 cm (4 cm) and both wavelengths. A power law fit was applied to data shown in Figs. 2(c), 2(d), 2(g) and 2(h), resulting in $R^{2}$ values higher than 0.999. In Figs. 2(e) and 2(f), horizontal lines indicating the 3% variation around the data mean value were added to highlight that the average noise amplitude is invariant with respect to $T_{\mathrm{meas}}$.

The results obtained by increasing $T_{\mathrm{meas}}$ can be summarized as follows: (i) the 1 Hz peak amplitude increases with $T_{\mathrm{meas}}$; (ii) the average noise amplitude is invariant with respect to $T_{\mathrm{meas}}$; and (iii) the 1 Hz peak contrast value increases with $T_{\mathrm{meas}}$.

#### 3.1.3 Effect of the sampling rate $f_{s}$

**Fig 3:**
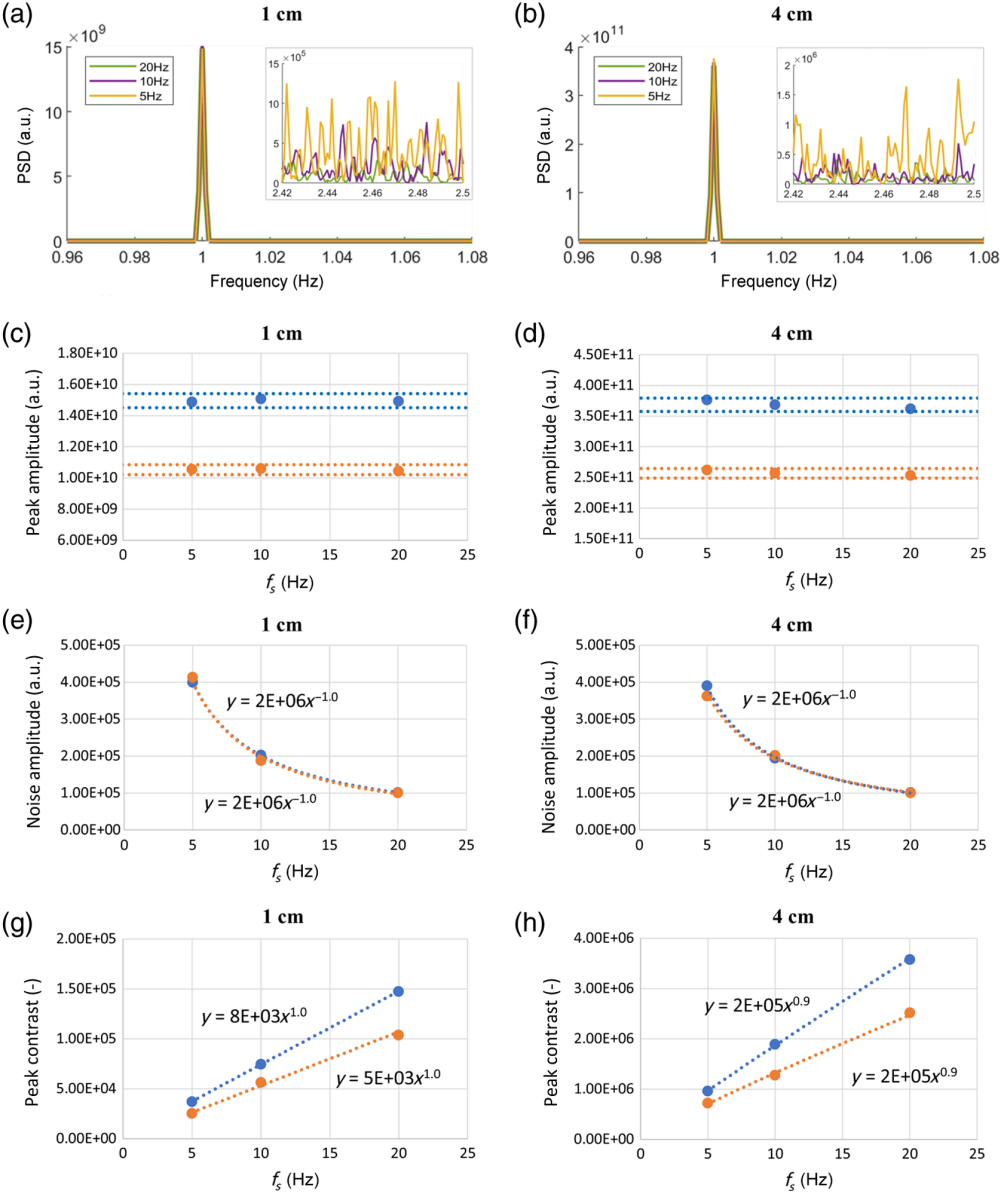
Effect of $f_{s}$ (case H_$f_{s}$). The two columns represent signals acquired with SSD of 1 cm (left) and 4 cm (right). (a), (b) Examples of PSDs obtained at 690 nm. The smaller boxes show a zoom of the PSD noise region. (c), (d) Amplitude of the spectral peak at 1 Hz; (e), (f) amplitude of the average noise; (g), (h) peak contrast. The results are reported for 690 nm, in blue, and 830 nm, in orange. A power law fit is applied to each dataset in panels (e), (f), (g), and (h), all giving $R^{2}\geq 0.99$. In panels (c) and (d), the horizontal lines represent a 3% variation around the mean value.

Figure 3 shows the effect of varying the $f_{s}$ on the spectra obtained in case H_$f_{s}$. Panels (a) and (b) show the PSD obtained for the SSD of 1 and 4 cm, respectively. Data are shown for a single wavelength (690 nm) as the results for the other wavelength (830 nm) are similar. The smaller box on the right of each graph represents a zoom on a higher-frequency region, showing the noise level in the three signals. Figures 3(c)–3(h) show the amplitude of the spectral peak at 1 Hz, the amplitude of the average noise, and the 1 Hz peak contrast as a function of $f_{s}$, for SSD = 1 cm (4 cm) and both wavelengths. In Figs. 3(c) and 3(d), horizontal lines indicating the 3% variation around the data mean value were added to highlight that the 1 Hz peak amplitude is invariant with respect to $f_{s}$. A power law fit was applied to the data shown in Figs. 3(e)–3(h), resulting in $R^{2}$ values higher than 0.99.

The results obtained by increasing $f_{s}$ can be summarized as follows: (i) the 1 Hz peak amplitude is invariant with respect to $f_{s}$; (ii) the average noise amplitude decreases with ${\left(f_{s}\right)}^{-1}$; and (iii) the 1 Hz peak contrast value increases with $f_{s}$.

#### 3.1.4 Comparison among different SSDs

**Fig. 4:**
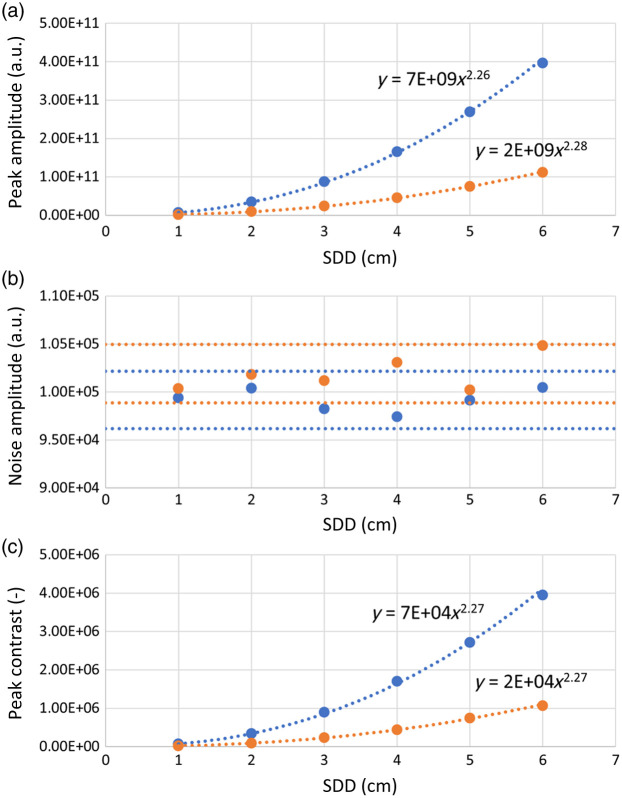
Effect of SDD (case H_SDD). (a) Amplitude of the spectral peak at 1 Hz, (b) amplitude of the average noise, and (c) peak contrast. The results are reported for 690 nm, in blue, and 830 nm, in orange. A power law fit is applied to each dataset in panels (a) and (c), all giving $R^{2}\geq 0.999$. In panel (b), the horizontal lines represent a 3% variation around the mean value.

Comparing the results obtained for the two SDD values in Secs. 3.1.1–3.1.3, an increase in the peak amplitude and contrast can be observed when increasing the SDD (Figs. 1–3). This effect is mainly due to the increase in the length of the average optical path traveled by the detected photons within the medium.^28^ To better understand the effect of the SDD on the retrieved PSDs, we performed additional simulations varying SDD (H_SDD). The relative results are shown in Fig. 4. Panels (a)–(c) respectively show the amplitude of the spectral peak at 1 Hz, the amplitude of the average noise, and the 1 Hz peak contrast as a function of SDD, for the two wavelengths. In Fig. 4(b), horizontal lines indicating the 3% variation around the data mean value were added to highlight that the average noise amplitude is invariant with respect to SDD. A power law fit was applied to the data shown in Figs. 4(a) and 4(c), resulting in $R^{2}$ values higher than 0.999.

The results obtained by increasing SDD can be summarized as follows: (i) the 1 Hz peak amplitude increases approximatively with the square of SDD; (ii) the average noise amplitude is invariant with respect to SDD; and (iii) the 1 Hz peak contrast value increases approximatively with the square of SDD.

### 3.2 Bilayer Medium

In this section, we investigate and compare the depth-selectivity enhancement approaches employed in CW and TD fNIRS. The time duration of measurements and the sampling frequency were selected based on the results presented in Sec. 3.1 ($T_{\mathrm{meas}}=15\;\min$ and $f_{s}=20\;\mathrm{Hz}$, respectively). The average total number of photon counts was varied as in Sec. 3.1.1, although most results presented here are referred to cases ${\overset{¯}{N}}_{\mathrm{tot}}=10^{6}\;\mathrm{ph}$, which presents the clearest contrasts, and ${\overset{¯}{N}}_{\mathrm{tot}}=10^{5}\;\mathrm{ph}$, the closest to real TD fNIRS *in-vivo* measurements with an SDD of 4 cm and a 20 Hz sampling rate, performed with a device previously developed at our department.^18^

#### 3.2.1 Time-windowing of the DTOFs

**Fig. 5:**
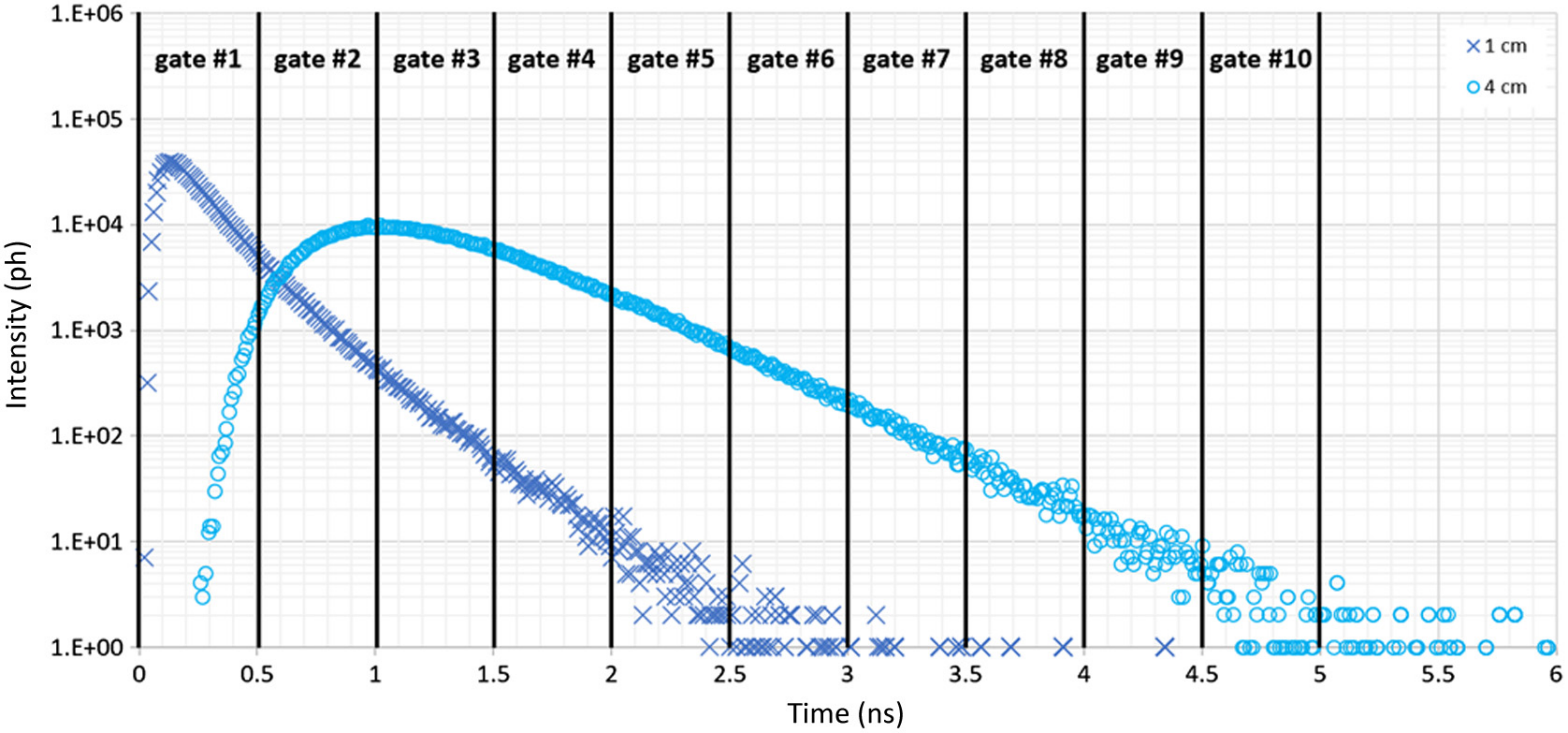
Example of DTOFs as recorded at SDD = 1 cm (dark blue, crosses) and SDD = 4 cm (light blue, circles) for ${\overset{¯}{N}}_{\mathrm{tot}}=10^{6}\;\mathrm{ph}$ and λ=690  nm. The applied time-windowing is represented by means of black vertical lines, positioned at the edges of each gate.

A time-windowing of the DTOFs was applied using 10 gates of 500 ps width, covering a 5 ns time window starting from the IRF temporal position (Fig. 5).

**Fig. 6:**
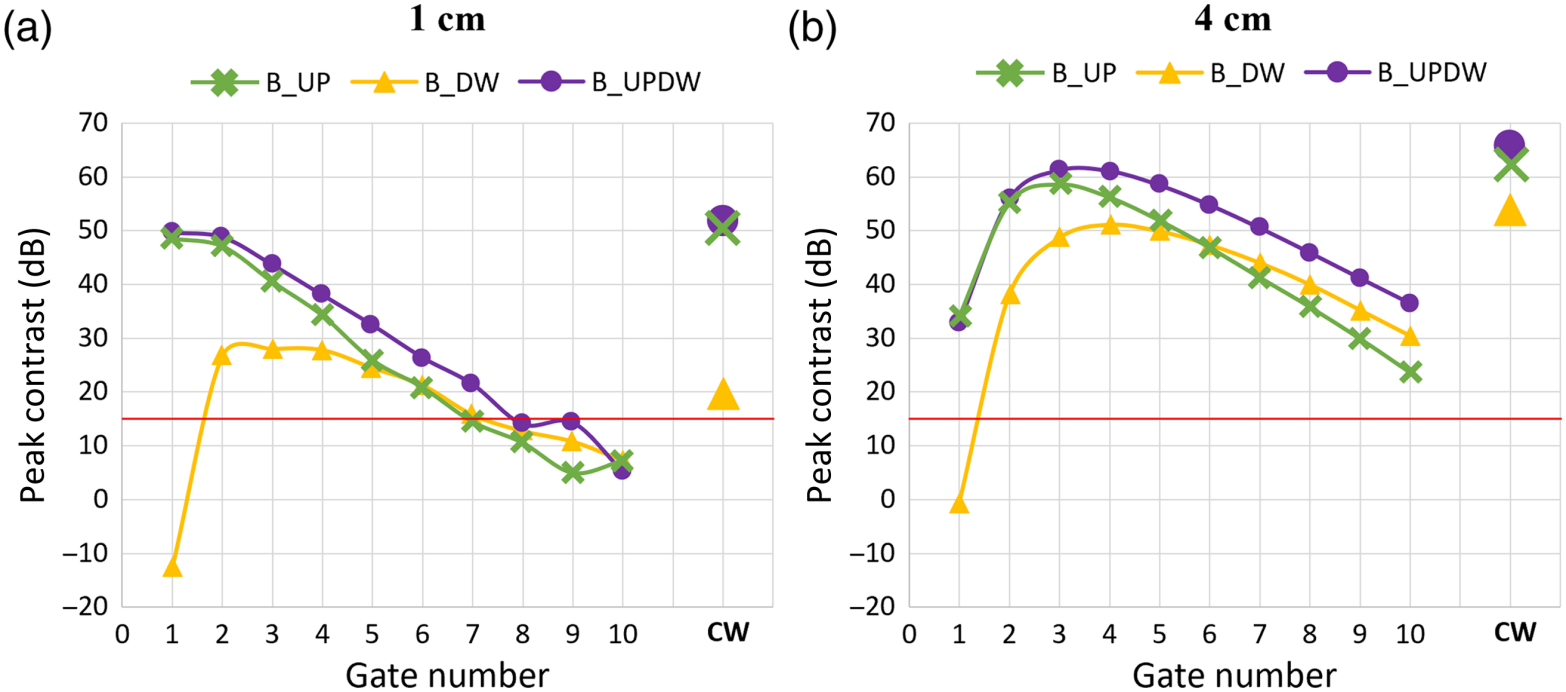
Contrast value of the 1 Hz peak as a function of the gate number for case B_UP (green, crosses), case B_DW (yellow, triangles), and case B_UPDW (purple, circles), with ${\overset{¯}{N}}_{\mathrm{tot}}=10^{6}\;\mathrm{ph}$. (a) Results obtained for SSD = 1 cm; (b) results obtained for SDD = 4 cm. In both graphs, the red horizontal line represents the peak significance threshold of 15 dB.

Figure 6 provides a comprehensive summary of the results obtained for cases B_UP, B_DW, and B_UPDW, showcasing the contrast of the 1 Hz peak in the PSD of the number of photon counts within each gate, as a function of the gate number, for ${\overset{¯}{N}}_{\mathrm{tot}}=10^{6}\;\mathrm{ph}$, both SDDs in Figs. 6(a) and 6(b), respectively, and a single wavelength (690 nm). The results are denoted with green crosses for case B_UP, with yellow triangles for case B_DW, and with purple circles for case B_UPDW. The results obtained for the CW fNIRS signals are also shown on the right. The contrast values are expressed in decibels, and the red horizontal line represents a peak significance threshold of 15 dB, tailored specifically for this analysis, which is the maximum contrast in the noise frequency range (Sec. 2.2.2).

Remarkably, a significant peak at 1 Hz was observed in the PSD of each gate that contained a not null portion of the reflectance curve (DTOFs retrieved for the short SDD decayed to zero around 3 ns, leaving the last four gates with only Poisson noise, see Fig. 5) for case B_UP (green crosses) and case B_UPDW (purple circles). This is a notable result because a significant peak is found even in gates covering the very end of the DTOF tail [from around gate #5 in Fig. 6(a) and gate #10 in Fig. 6(b)], in which the average number of photon counts is lower than 20 (Fig. 5). The only exception is represented by the first gate in case B_DW (yellow triangles), for both SDDs. In addition, the curves exhibit differences in peak and barycenter position and slope in the last gates. Another remarkable result is that the PSD calculated for the CW fNIRS signal always shows a substantial peak at the perturbation frequency. Of particular importance is the sensitivity shown by the CW fNIRS signal recorded at 1 cm to the perturbation imposed in depth [Fig. 6(a), case B_DW, yellow triangles], where, even if less pronounced, the peak at 1 Hz remains indeed significant.

#### 3.2.2 Estimated hemodynamic parameters

**Fig. 7:**
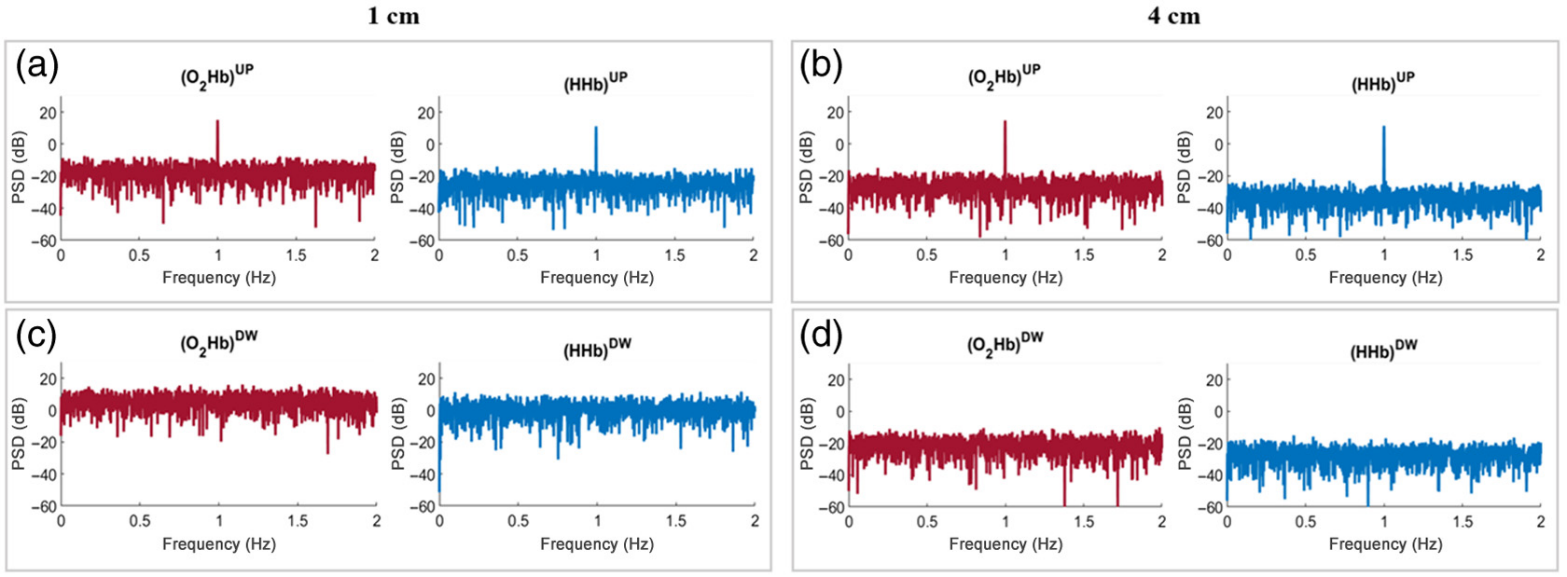
PSDs of the hemodynamic parameters obtained from TD fNIRS for case B_UP, with ${\overset{¯}{N}}_{\mathrm{tot}}=10^{5}\;\mathrm{ph}$. (a) Signals acquired at SDD = 1 cm, superficial layer (UP); (b) signals acquired at SDD = 4 cm, superficial layer; (c) signals acquired at SDD = 1 cm, deeper layer (DW); (d) signals acquired at SDD = 4 cm, deeper layer.

**Fig. 8:**
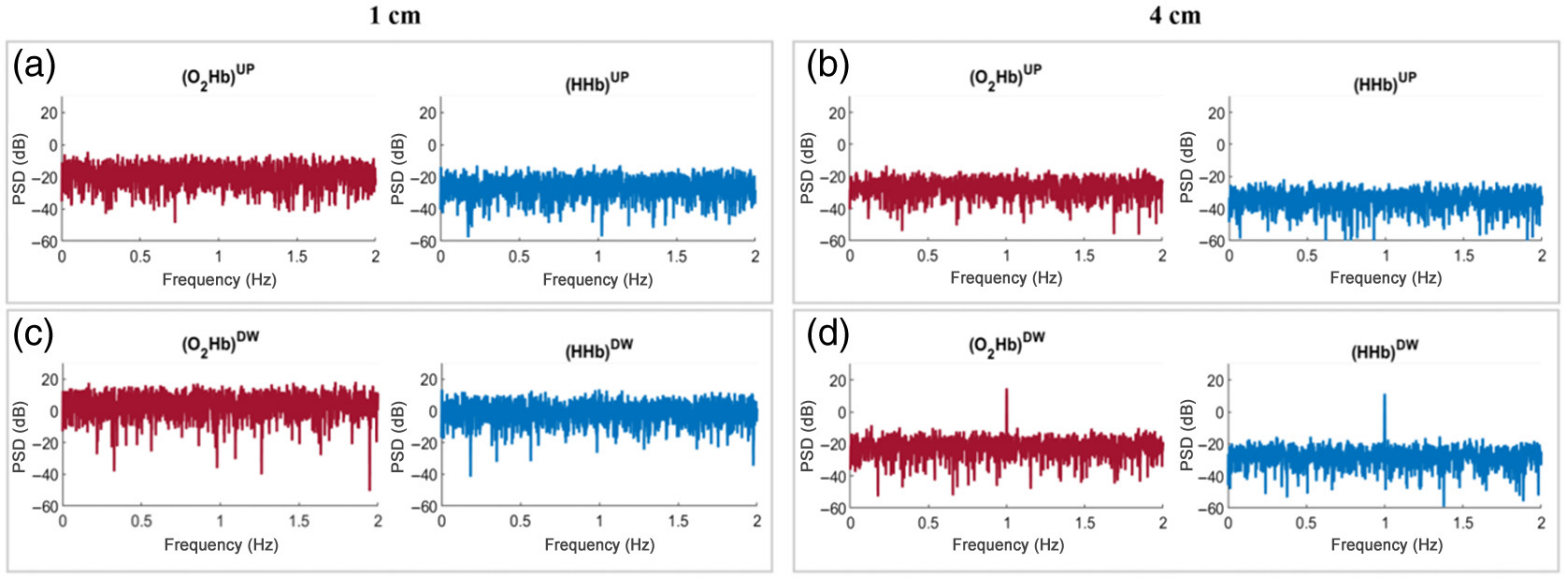
PSDs of the hemodynamic parameters obtained from TD fNIRS for case B_DW, with ${\overset{¯}{N}}_{\mathrm{tot}}=10^{5}\;\mathrm{ph}$. (a) Signals acquired at SDD = 1 cm, superficial layer (UP); (b) signals acquired at SDD = 4 cm, superficial layer; (c) signals acquired at SDD = 1 cm, deeper layer (DW); (d) signals acquired at SDD = 4 cm, deeper layer.

**Fig. 9:**
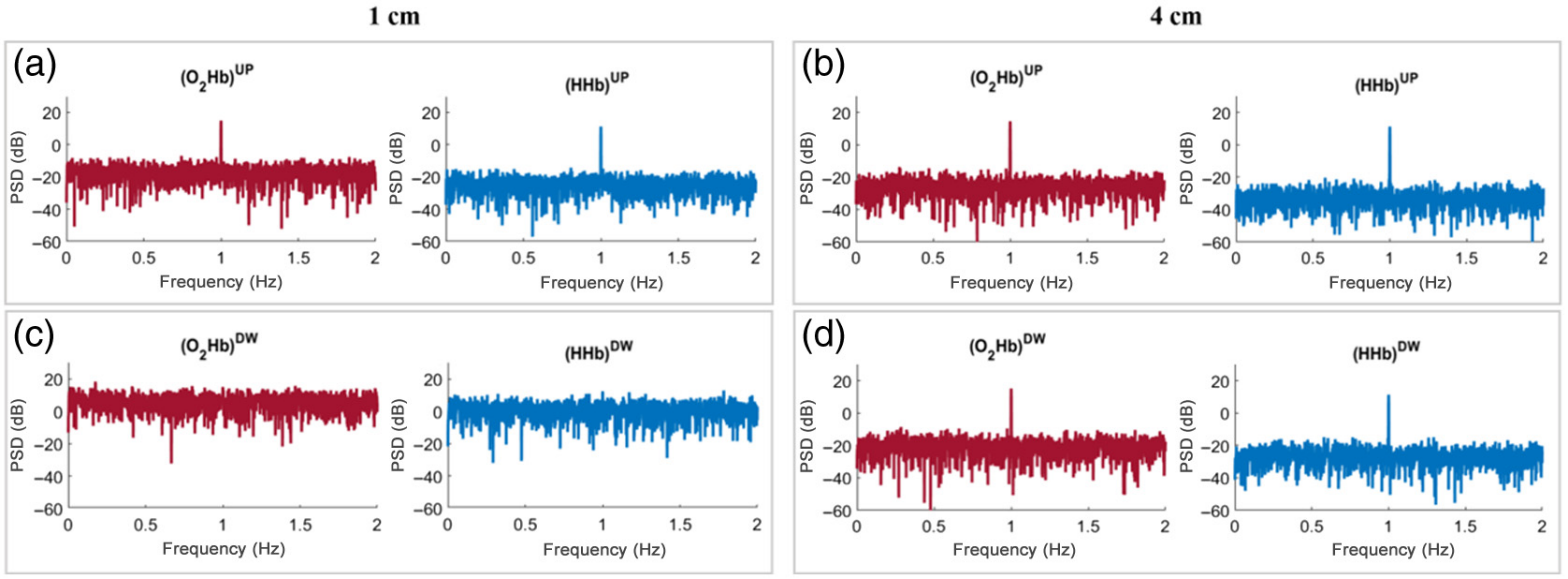
PSDs of the hemodynamic parameters obtained from TD fNIRS for case B_UPDW, with ${\overset{¯}{N}}_{\mathrm{tot}}=10^{5}\;\mathrm{ph}$. (a) Signals acquired at SDD = 1 cm, superficial layer (UP); (b) signals acquired at SDD = 4 cm, superficial layer; (c) signals acquired at SDD = 1 cm, deeper layer (DW); (d) signals acquired at SDD = 4 cm, deeper layer.

For TD fNIRS, the TMPP method was applied to retrieve the hemodynamic parameters in the two layers of the tissue. The obtained results are shown in Fig. 7 for case B_UP, in Fig. 8 for case B_DW, and in Fig. 9 for case B_UPDW. The results are presented for ${\overset{¯}{N}}_{\mathrm{tot}}=10^{5}\;\mathrm{ph}$ and both SDDs.

In case B_UP (Fig. 7), oscillations in the hemodynamic parameters of both layers were accurately reconstructed for each SDD as the PSD of both hemoglobin species exhibited a visible peak at the imposed perturbation frequency (1 Hz) in the superficial layer [UP, Figs. 7(a) and 7(b)], but not in the deeper layer [DW, Figs. 7(c) and 7(d)], as expected. In case B_DW (Fig. 8), similar results were observed for the long SDD [4 cm, Figs. 8(b) and 8(d)], with a visible peak at the perturbation frequency in the PSD of the hemoglobin concentrations calculated in the DW layer [Fig. 8(d)], but not in the UP layer [Fig. 8(b)], as expected. However, when the short SDD [1 cm, Figs. 8(a) and 8(c)] was used, different results were obtained. In this case, although the UP layer static behavior was correctly reconstructed [Fig. 8(a)], the perturbation peak in the PSD was no longer visible in the DW layer [Fig. 8(c)], where it should be present. The same observations regarding the lower sensitivity of short SDD measurements to variations in the deeper DW layer can also be done for case B_UPDW [Fig. 9(c)]. When considering SDD = 4 cm, Figs. 9(b) and 9(d) demonstrate a flawless reconstruction of the PSD for both species and both layers. The superimposition of the two contributions had no discernible effects, evident from the perfect overlap between PSDs in Figs. 7(b) and 9(b) [Figs. 8(d) and 9(d)]. Comparing the above PSDs, the amplitude of the 1 Hz peak is indeed equal for both species, except for the variability due to the background noise. Analogous results were obtained by imposing oscillations with different frequencies and/or amplitudes in the two layers of the medium (cases B_Amp, B_Freq, and B_AmpFreq) while considering them either in-phase or out-of-phase (data not shown).

**Fig. 10:**
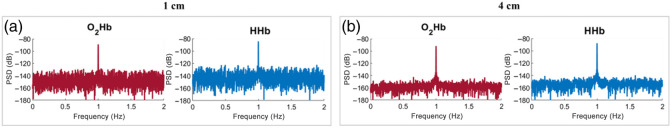
PSDs of the hemodynamic parameters obtained from CW fNIRS for case B_UP, with ${\overset{¯}{N}}_{\mathrm{tot}}=10^{6}\;\mathrm{ph}$. (a) Signals acquired at SDD = 1 cm; (b) signals acquired at SDD = 4 cm.

**Fig. 11:**
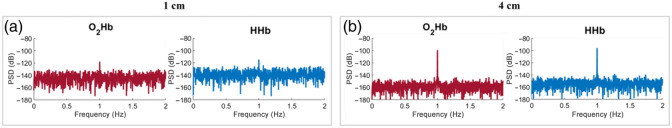
PSDs of the hemodynamic parameters obtained from CW fNIRS for case B_DW, with ${\overset{¯}{N}}_{\mathrm{tot}}=10^{6}\;\mathrm{ph}$. (a) Signals acquired at SDD = 1 cm; (b) signals acquired at SDD = 4 cm.

**Fig. 12:**
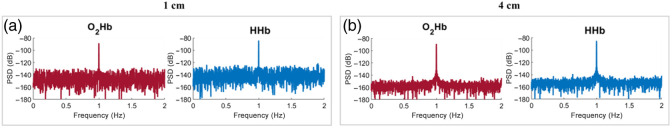
PSDs of the hemodynamic parameters obtained from CW fNIRS for case B_UPDW, with ${\overset{¯}{N}}_{\mathrm{tot}}=10^{6}\;\mathrm{ph}$. (a) Signals acquired at SDD = 1 cm; (b) signals acquired at SDD = 4 cm.

For CW fNIRS, the results are shown in Fig. 10 for case B_UP, Fig. 11 for case B_DW, and Fig. 12 for case B_UPDW. The results are shown for ${\overset{¯}{N}}_{\mathrm{tot}}=10^{6}\;\mathrm{ph}$ to account for the higher intensities usually characterizing CW fNIRS signals compared with TD fNIRS ones.

In all of the above cases, a visible peak is present at the perturbation frequency for both species and both SDDs. This is particularly interesting in case B_DW (Fig. 11) when the perturbation is imposed solely in the DW layer. In Fig. 11, the CW fNIRS signals recorded at both SDDs show sensitivity to the perturbation imposed in the DW layer of the medium for both species. In Sec. 3.2.1, the sensitivity shown by the signal recorded at 1 cm [Fig. 11(a)] is a notable and unexpected result.

## 4 Discussion

In this work, we conducted a simulation study to assess the viability of employing TD fNIRS for the examination of cerebral hemodynamic oscillations during resting-states in humans. Previous research has delved into this area using fMRI, CW fNIRS, and FD fNIRS techniques, but the adoption of TD fNIRS has been limited by lower SNRs. As highlighted in Sec. 1, to the best of our knowledge, only two attempts of using TD fNIRS in this field of investigation are reported in the literature,^13^^,^^14^ and no studies that systematically evaluate the sensitivity of TD fNIRS to periodical perturbations in the optical properties of the probed medium exist. The aim of our study was to fill an existing literature gap, providing some guidelines for researchers to design optimal experimental protocols when investigating cerebral hemodynamics in humans using fNIRS, contributing to addressing the crucial challenge of standardizing fNIRS experimental procedures. To this purpose, we performed numerical simulations of TD and CW fNIRS acquisitions, considering two different geometries of the probed medium using homogeneous and bilayer models.

### 4.1 Homogeneous Medium

Exploiting the homogeneous model, the influence of the number of detected photons per DTOF (${\overset{¯}{N}}_{\mathrm{tot}}$), the overall measurement length ($T_{\mathrm{meas}}$), sampling frequency ($f_{s}$), and the used SDD on the recorded signal PSD were tested. The sensitivity of fNIRS to sinusoidal perturbations in the hemodynamic parameters of the medium was expressed in terms of an ad-hoc defined spectral peak contrast, calculated from the PSD of the intensity signal. Due to this choice, the reported results are valid for both TD and CW fNIRS.

The results show that the contrast value obtained with the two techniques increases proportionally with ${\overset{¯}{N}}_{\mathrm{tot}}$, $T_{\mathrm{meas}}$, and $f_{s}$ and approximatively with the square of SDD, when the parameters are varied independently. The same relationships were found to be valid when using different values of perturbation amplitude (0.5% and 5%, data not shown). These findings, together with the successive evaluation of different data analysis pipelines, provide insights into the optimization of fNIRS experimental designs. Depending on the specifications of the used instrument and the characteristics of the investigated phenomena, different combinations of acquisition parameters can be selected to maximize TD and CW fNIRS sensitivity. As an example, when working with an instrument limited in power, for which the achievable ${\overset{¯}{N}}_{\mathrm{tot}}$ is not sufficient to obtain a clear spectrum, this limitation can be compensated for by increasing the value of $T_{\mathrm{meas}}$. Regarding $f_{s}$, any increase in this parameter will result in a decrease in the maximum number of photons detected (${\overset{¯}{N}}_{\mathrm{tot}}$) when the maximum allowable light intensity is used. However, in TD fNIRS, sampling at higher frequencies is particularly useful when using depth-selectivity algorithms based on the time-windowing of the DTOF. The use of corrective methods such as the TMPP reduces the effect due to changes in ${\overset{¯}{N}}_{\mathrm{tot}}$ on the PSD of the derived signal, while the effects of $T_{\mathrm{meas}}$ and $f_{s}$ remain unchanged (data not shown). When the desired effect is to maximize the contrast in the PSD of $O_{2}\mathrm{Hb}$ and HHb of the two layers as calculated using the TMPP method, increasing $f_{s}$ is therefore an effective method even at the expense of the detected number of photons. Once the minimum threshold for the coefficient of variation^35^ and the corresponding one for ${\overset{¯}{N}}_{\mathrm{tot}}$ are set,^18^ the best choice to optimize the quality of the obtained PSD is to use the maximum $f_{s}$ that guarantees staying above it.

### 4.2 Bilayer Medium

The bilayer model was chosen for investigating the potentiality of the depth-selectivity enhancement approaches employed in TD fNIRS compared with the multi-distance CW fNIRS approach.

First, a time-windowing of the DTOFs was used to evaluate the depth-selectivity of TD fNIRS measurements.^15^ This approach is based on the evidence that, in TD fNIRS, photons detected with a longer time of flight show a higher probability of having traveled deeper into the medium compared with those detected shortly after the pulse injection.^15^ Thus, it can be used to isolate superficial and deeper contributions using a single-channel measurement. For this reason, it represents an alternative to the short-SDD regression method used in CW fNIRS, mitigating the challenges associated with the use of multiple SSDs.^21^ To evaluate the potentiality of the time-windowing approach, the signals obtained in cases B_UP, B_DW, and B_UPDW were analyzed by slicing the DTOF using 10 gates, each with a width of 500 ps, covering the time window between 0 and 5 ns following the IRF temporal position. The PSDs of the number of photons detected within each gate were calculated and compared between the two SDDs and among the three cases.

The results indicate that, irrespective of the SDD, photons detected within the first 500 ps are affected by the variation of the optical properties of the medium only when they occur in the superficial layer (Fig. 6, gate 1), confirming that these photons do not penetrate at a depth greater than 1 cm in the tissue (i.e., a typical thickness of the scalp). As a result, the shape of the DTOF in this time region is not influenced by the in-depth behavior of the medium. Consequently, the fNIRS signal collected within the first 500 ps from the pulse delivery contains information solely on the oscillatory pattern in the UP layer. By considering narrower gate widths and non-ideal IRF,^18^ we also verified that the information content relative to the UP layer remains confined within a time window of ∼500 to 600 ps from the IRF temporal position (data not shown). Then, the simple gating approach applied to TD fNIRS measurements can effectively identify oscillations originating in the superficial layer. This result opens the way for the implementation of a regression method for the TD fNIRS signal that mirrors the approach used in the CW modality, with the additional advance of removing the requirement for multiple SDD acquisitions.^21^

Another remarkable result obtained with these simulations is the high sensitivity of the TD fNIRS signal to periodical perturbations in the optical properties of the probed medium. This is demonstrated by the presence of a significant spectral peak in PSDs associated with time windows covering the very end of the DTOF tail, where the detected photons are on the order of some tens or less. After this first analysis phase, two different methods were applied to derive the hemodynamic parameters in the tissue.

For TD fNIRS, the TMPP method was used. When the perturbed layer was the superficial one (case B_UP), the PSD of the hemodynamic parameters of both layers was accurately reconstructed for each SDD (1 and 4 cm), as the PSD of both hemoglobin species exhibited a visible peak at the imposed perturbation frequency solely in the UP layer (Fig. 7). Similar results were observed for the long SDD when the perturbed layer was the deeper one (case B_DW), with a visible peak at the perturbation frequency in the PSD of the hemoglobin concentrations in the DW layer, but not in the UP layer, as expected [Figs. 8(b) and 8(d)]. Conversely, different results were obtained for the signal recorded at the short SDD in the same case. Although the UP layer static behavior was correctly reconstructed, the perturbation peak in the PSD was no longer visible in the DW layer, where it should have been present [Figs. 8(a) and 8(c)]. The absence of this peak is attributed to the reduced number of photons that reached the deeper region of the medium, known as *late* photons. Despite the number of photons used to construct the DTOFs being the same for both SDDs, the distribution shape differs, being narrower for the shorter SDD (Fig. 5). Consequently, although the absolute number of *late* photons increases at shorter SDD,^36^ the ratio between the *late* and *early* photons, which traveled in the more superficial region of the medium and hence occupy the first portion of the DTOF, is much lower for the short compared with the long SDD.^36^ This disparity in photon distribution reduces the TD fNIRS signal sensitivity to the deeper layer of the medium relative to the superficial one, limiting its ability to detect the perturbation applied in case B_DW.

Additional simulations were performed by imposing a perturbation of reduced amplitude (0.75%, 0.5%, 0.25%, and 0.10% of the baseline values) only in the deeper layer. The obtained results (not shown) were analogous to those discussed in Sec. 3.2.1, even for the lower value of ${\overset{¯}{N}}_{\mathrm{tot}}$ ($10^{4}\;\mathrm{ph}$). The above results demonstrated that, with an adequate SDD, the use of the TMPP method allows us to accurately retrieve the hemoglobin concentration signals from both layers of the medium. Consequently, TD fNIRS has the capability to detect and precisely locate periodical perturbations in the hemodynamic parameters within the probed medium using a single-point measurement approach.

A final set of simulations was designed to evaluate the effects generated by the superimposition of multiple Fourier contributions originating in the two layers of the probed medium, having different amplitudes and/or frequencies (B_Amp, B_Freq, and B_AmpFreq). The PSDs obtained for each hemodynamic parameter and layer were compared with the analogous ones obtained when imposing a single oscillatory perturbation in the medium. No significant deviation was found among PSDs obtained for the layer having the same oscillation imposed, independently from the behavior or the other one. As an example, comparing the UP layer in cases B_UP, B_UPDW, and B_Amp, in which the same oscillation is imposed (1% amplitude, 1 Hz frequency), the resulting PSDs are perfectly superimposable (excluding the contribution of noise) at the frequency of interest. The same result is obtained when comparing the DW layer in cases B_DW, B_UPDW, and B_Freq. This result indicates that the TMPP method can be effectively used to identify the distinct oscillatory components in each layer, without any interference or cross-contamination between them. Similar to what was previously done, the above simulations were also repeated, introducing the convolution with a real IRF.^18^ As before, the resulting PSD showed no significant differences, confirming the robustness of the TMPP method.

However, the TMPP approach is limited by the need for an *a priori* accurate description of the probed medium geometry. Moreover, as the model complexity increases, the computational load escalates rapidly, necessitating simplifications when dealing with measurements on multi-layered structures such as the human brain. Inaccuracies in the medium description (typically the unknown thickness of the UP layer) introduce errors in the calculation of the hemodynamic parameters and restrict the method’s capability to precisely discern the hemodynamic behavior in the different regions of the medium. In this context, exploring the depth-selectivity of the TD fNIRS signal using the time-windowing approach could represent an interesting alternative.

### 4.3 CW and TD fNIRS

To allow for a direct comparison between TD and CW fNIRS data, DTOFs were numerically integrated over time to obtain the measured light intensity. For CW fNIRS, the hemoglobin concentrations were then recovered from these signals by exploiting the modified Lambert–Beer law. Different from TD, CW fNIRS does not allow for the separation of the information about the oscillations associated with the two layers of tissue using a single SSD. Depth selectivity in this configuration relies on the comparison of the different signals measured simultaneously at different SDDs.^37^^,^^38^ In CW fNIRS studies, one or more short SDD channels are used as a regressor for signals recorded at longer SDDs to correct the latter for the superficial contributions. This approach does not consider the variation in fNIRS signal sensitivity to the different layers of the probed medium when the SDD is varied,^21^ and the conventional assumption underlying the multi-distance approach is that the penetration depth of photons detected at a given SDD in CW modality is limited to around a half of the SDD value.^21^^,^^37^ However, this assumption is challenged by the results of our study, in which the CW fNIRS signal recorded at 1 cm is affected by perturbations imposed in the DW layer of the medium. Significant peaks attributed to perturbations introduced in the deeper layer are indeed observable in the PSD of both the intensity and hemoglobin concentration signals [Fig. 11(a)]. This effect is probably due to the high sensitivity of fNIRS signals to periodical fluctuations even in the case of very few photons being affected. Owing to this unexpected sensitivity of the CW fNIRS signal at 1 cm to the perturbation in the DW layer, the existing short-distance correction methodologies when a Fourier-domain analysis of the signal is performed are worth re-evaluating. However, the relatively low amplitude of the 1 Hz peak in Fig. 11(a), coupled with the simplifications applied to the imposed perturbation, the measurement system, and the tissue model, suggests that the higher noise levels expected in real-world applications should prevent a significant impact of this effect on actual *in-vivo* measurements.

### 4.4 Limitations and Future Developments

The main limitations of this study lie in the idealized nature of the measurement system and of the perturbations applied and in the simplifications imposed on the geometrical model.

In our simulations, the measurement system was considered ideal, generating a delta-shaped IRF and null background noise. Although the broadening of the IRF demonstrated to have no significant effects on the obtained results (as discussed above), the effect of the background noise was not investigated. In real applications, this could lead to lower SNRs in the obtained PSDs, limiting the ability of the technique to detect low-amplitude oscillations.

Moreover, the sinusoidal perturbations were modeled with a constant amplitude and frequency, whereas throughout measurements in *in-vivo* applications, the perturbations of hemoglobin concentrations are expected to exhibit some level of variability due to the natural fluctuations of underlying physiological phenomena. The presence of frequency and phase variations may result in spectral peaks broadening or bifurcation, alongside a concurrent reduction in peak amplitude while maintaining a constant signal energy. These effects introduce complexity into the precise identification of individual spectral peaks and the subsequent classification based on the underlying phenomena.

Nonetheless, the limitations arising from the idealized nature of the measurement system and perturbations employed in our study do not invalidate the fundamental findings and conclusions presented thus far. Specifically, our findings on the optimal selection of the main operational parameters and the technique’s efficacy in localizing the detected perturbations within the sample layers remain valid.

Finally, we considered the probed medium either as homogeneous or composed of two homogeneous layers, which may not fully represent the complexity of real biological tissues. A multilayer model with a scalp layer, a bone layer, a cerebrospinal fluid layer, a vascular layer on the cerebral cortex, and a brain tissue layer would be necessary to represent more realistically the probed volume. Consequently, the sensitivity of TD fNIRS to fluctuations occurring *in vivo* could be lower than the one observed in this study.

As part of future work, tests will be conducted to assess the performance of TD fNIRS in *in-vivo* applications. In addition, further simulations will be employed to investigate the impact of inaccuracies in the model used to describe the probed medium when applying the TMPP correction.

## 5 Conclusions

In summary, this paper aimed at contributing to the emerging field of the study of brain hemodynamic oscillations through fNIRS. In particular, we focused on the oscillations during resting-state acquisitions with TD and CW fNIRS. Through a series of numerical simulations, we provided valuable guidelines for optimizing four main operational parameters (average photon count rate, measurement duration, sampling frequency, and SSD) to maximize the sensitivity of fNIRS to periodical perturbations of the medium optical properties and for defining the best experimental protocol for detecting resting-state cerebral hemodynamic oscillations. By leveraging the advantages of TD fNIRS, we demonstrated that this technique allows for the detection and depth-localization of periodical fluctuations occurring in the concentrations of $O_{2}\mathrm{Hb}$ and HHb within the probed medium using a single-point acquisition, offering an alternative to multi-distance CW fNIRS setups.

Our results demonstrate that the time-windowing of the DTOF is a suitable method for detecting the presence of oscillatory components in the signal, even in the presence of very few detected photons, together with the ability to isolate the behavior of the more superficial layer of the medium. Therefore, the implementation of a method that makes use of the first portion of the DTOF to correct the information encoded in the late photons could allow for obtaining a depth-selective description of the medium hemodynamics in the Fourier domain, even when its geometry is unknown or the photon count rate is insufficient to accurately retrieve the hemodynamic parameters. Moreover, our findings confirm that the use of the TMPP method to retrieve the $O_{2}\mathrm{Hb}$ and HHb concentrations allows us to correctly reconstruct the signals coming from the two layers of the medium with good sensitivity and no cross-talk.

## Data Availability

Data underlying the results presented in this paper are not publicly available but may be obtained from the corresponding authors upon reasonable request.
